# GLP-1 receptor agonism results in reduction in hepatic ethanol metabolism

**DOI:** 10.1038/s44324-025-00077-y

**Published:** 2025-09-18

**Authors:** Frhaan Zahrawi, Arumugam Suyavaran, Bubu A. Banini, Wajahat Z. Mehal

**Affiliations:** 1 The Section of Digestive Diseases, Yale School of Medicine, New Haven, CT USA; 2 West Haven Veterans Medical Center, New Haven, CT USA

**Keywords:** Hepatology, Liver diseases

## Abstract

Glucagon-like peptide 1 receptor (GLP-1R) agonists are used along with ethanol consumption, but their interactions are not understood. Our aim was to determine the effects of GLP-1R agonism on the liver in mouse models of high ethanol consumption. We identified that GLP-1R agonism reduced ethanol consumption, mitigated ethanol-induced upregulation of several liver metabolizing enzymes, including Cyp2e1 and also reduced Cyp2e1 independent of ethanol intake. As expected from a reduction in Cyp2e1, GLP-1R agonism resulted in increased blood ethanol levels. This occurred after a single dose of ethanol when given by gavage, and by the intraperitoneal route. This suggests that GLP-1R agonism can reduce ethanol-mediated hepatotoxicity despite continued ethanol consumption and elevate blood alcohol levels.

The approval of GLP-1R agonists for obesity and diabetes mellitus has resulted in higher demand and prescription for these indications, and a GLP-1R agonist is currently in a phase 3 clinical trial in metabolic dysfunction-associated steatohepatitis (MASH)^1^. The main mechanism of the therapeutic effect of GLP-1R agonists in obesity is considered to be via reduced calorie intake, and this is also considered to be true for their therapeutic effects on MASH^2^. Ethanol has a novel place in nutrition because it has some nutritional value, but with increased dosage, it precipitates a transition towards several pathologies, including ethanol-induced liver disease (ALD)^3,4^. MASH and ALD share several similarities, including excess intake of nutrients or ethanol, and the pathological features of hepatic steatosis, inflammation and fibrosis^5,6^. These similarities raise the important question of the potential therapeutic and any other effects of GLP-1R agonists in ALD and ethanol metabolism more broadly. Analogous to the well-documented ability of GLP-1R agonists to reduce appetite and food intake, there is increasing data on their ability to reduce ethanol intake in patients with AUD^7,8^. GLP-1R agonist-mediated reduction in ethanol consumption can be expected to improve ALD, and early data support this^9,10^. Hepatocytes have no or very low levels of GLP-1R, and studies report no direct effects of GLP-1R agonist on hepatocytes^11^.

Our aim was to determine the effects of systemic GLP-1R agonism on the liver. We employed the standard model of ALD in mice, extending the free access to ethanol consumption for 16 days, with up-titration of GLP-1R agonist semaglutide given subcutaneously for the first 7 days (up to a full dose of 30 nmol/Kg body weight/day via subcutaneous injection)^12^. Four experimental groups were segregated as follows: ethanol with and without GLP-1R agonism, and control liquid diet with and without GLP-1R agonism (Supplementary Fig. 1A). At day 16, the livers were examined for steatosis, changes in hepatic expression of genes involved in de novo lipogenesis, oxidant stress and inflammation. As expected, GLP-1R agonism resulted in a reduced intake of both control liquid and ethanol diets, and a corresponding reduction of body weight in both the control and ethanol groups (Supplementary Fig. 1B). Compared to the control diet, the ethanol diet resulted in a very significant increase in hepatic steatosis (perilipin 2 staining) and this was significantly less in the GLP-1R agonist-treated ethanol diet group (Fig. 1A, B). The reduction in steatosis in the GLP-1R agonist-treated group was accompanied by suppressed expression of hepatic genes for de novo lipogenesis (*Acc1*, *Srebp1c* and *Fasn*), for reactive oxygen stress (*Nrf2*, *Nox2*) and inflammation (*Mpo*, *Il-6*, *Il-12*) (Fig. 1C–E). To confirm if the reduction in oxidative stress was biochemically significant, we quantified lipid peroxidation by immunostaining for 4-hydroxynonenal (4HNE) and observed a significant increase in hepatic 4HNE in mice on ethanol, which was suppressed by GLP-1R agonist treatment (Fig. 1F, G). These data demonstrate that in mice, GLP-1R agonism results in a reduction in regular nutrient and ethanol diet intake, with less steatosis, de novo lipogenesis, inflammation and oxidative stress. To better understand the intersection between the hepatic effects of GLP-1R agonist and ethanol, we compared changes in the hepatic transcriptome by GLP-1R agonist with the changes induced by ethanol alone. We identified two gene sets. Set X (921 genes) was altered by GLP-1R agonism in both the control and the ethanol diet, but not by ethanol alone. Set Y (756 genes) was altered by GLP-1R agonism in both the control and the ethanol diet, and also regulated by ethanol in the absence of a GLP-1R agonist (Supplementary Fig. 2A). We expected set X to be enriched in GLP-1R agonism regulated genes and set Y to be enriched in GLP-1R agonism regulated genes and genes also regulated by ethanol. When examined by molecular function and biological process analysis, set X and set Y were both found to contain genes involved in a broad set of pathways (Supplementary Fig. 2B–G). The set of genes central to hepatic ethanol metabolism (alcohol dehydrogenase, aldehyde dehydrogenase and cytochrome P450) clustered almost entirely in sets X and Y (Supplementary Fig. 3B–D). It is known that ethanol consumption upregulates many of the genes involved in ethanol metabolism, and since GLP-1R agonism reduced ethanol consumption, it was expected that GLP-1R agonism would indirectly reduce expression of these genes^13^. It was, however, interesting to note that many of the ethanol- metabolizing genes regulated by GLP-1R agonism were in group X, which contains the genes that are not altered by ethanol. This demonstrates that GLP-1R agonism also regulates (mostly reducing) ethanol-metabolizing genes, which are independent of ethanol intake.

**Fig. 1:**
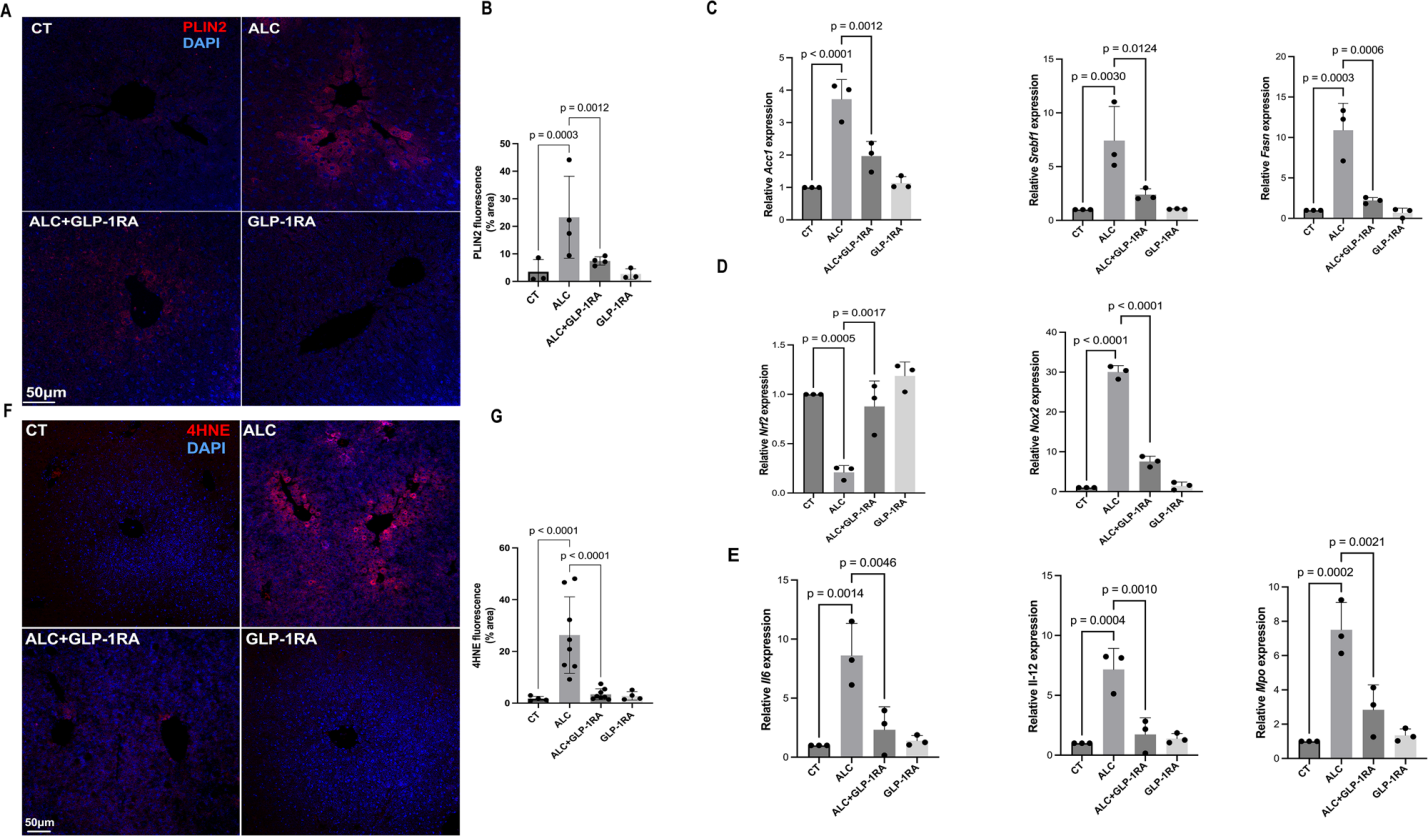
GLP-1R agonism reduces hepatic steatosis, inflammation and redox injury. **A**, **B** Immunofluorescence staining for lipid marker PLIN2 in liver sections (scale = 50 µm) with quantification performed using ImageJ. At least five images were analyzed per sample. Sample size: CT and CT + GLP-1R *N* = 3; ALC and ALC + GLP-1R *N* = 4. Data were presented as mean ± SD, with statistical analysis by two-way ANOVA with Holm–Šídák’s test. **C** qPCR analysis of key lipogenesis-related genes, including *Acc1, Sreb1f*, and *Fasn*. Data presented as mean ± SD; sample size: *n* = 3 biologically independent samples. Statistical comparisons were made using one-way ANOVA with Dunnett’s test. **D** qPCR analysis of genes associated with oxidative stress, including *Nrf2 and Nox2*. Data presented as mean ± SD; sample size: *n* = 3 biologically independent samples. Statistical comparisons were made using one-way ANOVA with Dunnett’s test. **E** qPCR analysis of inflammatory genes, including *Il-6, Il-12 and Mpo*. Data presented as mean ± SD; sample size: *n* = 3 biologically independent samples. Statistical comparisons were made using one-way ANOVA with Dunnett’s test. **F**, **G** Immunofluorescence staining for 4-hydroxynonenal (4HNE) in liver sections (scale = 50 µm), with quantification performed using ImageJ. At least five images were analyzed per sample per group. Sample size: CT and CT + GLP-1R *N* = 4; ALC and ALC + GLP-1R *N* = 8. Data were shown as mean ± SD, with statistical analysis by two-way ANOVA with Holm–Šídák’s test.

Hepatocyte Cyp2e1 plays a major role in ethanol metabolism, generating high levels of ROS, which can damage hepatocytes^13^. Bulk RNA-Seq data suggested reduced levels of *Cyp2e1* in mice treated with GLP-1RA when given free access to a regular diet or a liquid ethanol diet (Supplementary Fig. 3B). Importantly, we confirmed that hepatocyte Cyp2e1 protein levels were significantly lower in the presence of GLP-1R agonism in both the control and ethanol consumption group (Fig. 2A, B)^13^. To test if GLP-1R agonism can reduce *Cyp2e1* mRNA and protein levels independently of ethanol consumption, we examined the effect of GLP-1R agonism in the absence of food and ethanol intake. After a single subcutaneous dose of the GLP-1R agonist semgalutide (30 nmol/Kg), mice were given free access to water but no food or ethanol. After 16 h, the livers were examined for mRNA and protein levels of CYP2E1. There was a significant reduction in *Cyp2e1* gene expression and protein levels in the GLP-1R agonist group compared to the control (Fig. 2C, D). The fact that GLP-1R agonism can reduce *Cyp2e1* levels independent of ethanol intake suggests that GLP-1R agonism may reduce ethanol metabolism and, therefore, result in higher serum ethanol levels. To test if GLP-1R agonism can increase blood ethanol levels, a fixed dose of ethanol (2.5 g/kg) was administered by a single gavage or intraperitoneally to C57BL/6N mice, and serum ethanol concentration was measured at 30, 90, 150, 270, and 330 min. GLP-1R agonism resulted in significantly elevated blood ethanol levels at 30, 90, and 150 min after ethanol gavage in male and female mice, compared to ethanol gavage alone (Fig. 2E, F). Intraperitoneally administered ethanol also resulted in higher plasma concentrations in the presence of GLP-1R agonism, demonstrating that the ethanol-metabolizing enzymes present in the intestinal epithelium are not major contributors to this GLP-1R agonism effect on blood ethanol levels (Fig. 2F).

**Fig. 2:**
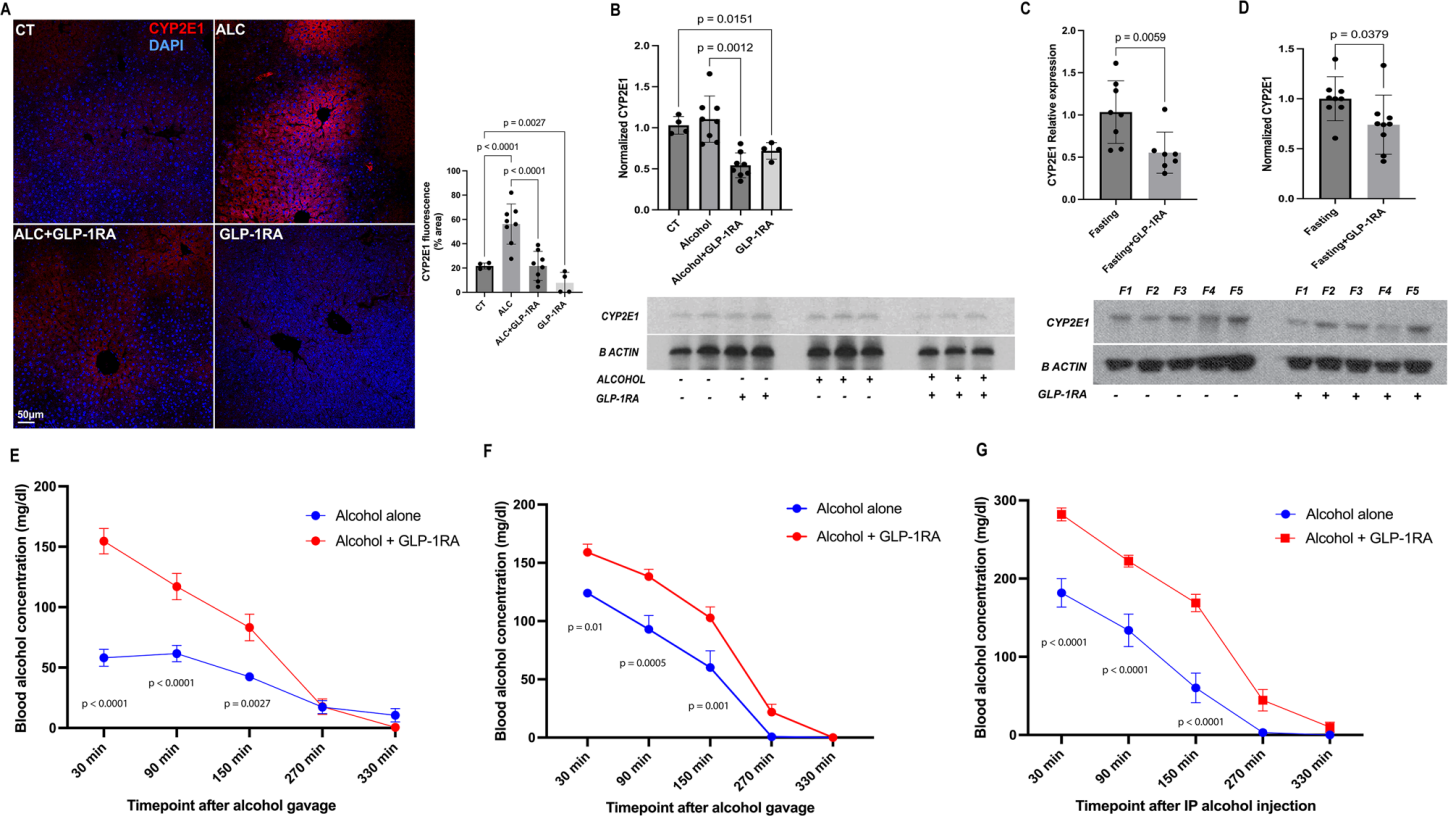
GLP-1R agonism results in reduced liver levels of Cyp2e1, and higher blood levels of ethanol. **A** Immunofluorescence staining for Cyp2e1 in liver sections (scale = 50 µm). Quantification of fluorescence intensity was performed using ImageJ, with five images analyzed per sample. sample size: CT and CT + GLP-1R *N* = 4; ALC and ALC + GLP-1R *N* = 8. Data were presented as mean ± SD; statistical analysis by two-way ANOVA with Holm–Šídák’s test. **B** Western blot analysis of Cyp2e1 protein expression in ethanol study, sample size: CT and CT + GLP-1R *N* = 4; ALC and ALC + GLP-1R *N* = 8. Statistical analysis was done using Brown–Forsythe and Welch’s ANOVA tests. Data were shown as mean ± SD. **C** Relative mRNA expression of *Cyp2e1* was measured by qPCR in the fasting group vs. fasting + GLP-1R group. Mice were fasted for 16 h after GLP-1R agonist administration to control for oral intake. Statistical analysis by (Mann–Whitney *U*-test). Data were shown as mean ± SD. Sample size: fasting group *N* = 8; fasting + GLP-1R *N* = 7. **D** Western blot analysis of Cyp2e1 protein expression in the fasting experiment, with quantification as mean ± SD. Statistical analysis by (Mann–Whitney *U*-test). Data were shown as mean ± SD—data presented from eight independent samples for each group (*n* = 8). **E** Blood ethanol concentration over time following a single subcutaneous dose of GLP-1R agonist (30 nmol/kg) or saline, administered the night before ethanol gavage (2.5 g/kg). Blood samples were collected at 30, 90, 150, 270, and 330 min post-gavage. Sample sizes: *n* = 5 for ethanol (gavage) + saline group, *n* = 4 for ethanol(gavage) + GLP-1R agonist group. Group. Statistical analysis by two-way ANOVA with Holm–Šídák’s test showed a significant *P* < 0.001 difference between the groups at times (30, 90, 150 min *p* = 0.002) with a mean difference of −36 (SE 8.5). This was performed on male mice. **F** Blood ethanol concentration over time following a single subcutaneous dose of GLP-1R agonist (30 nmol/kg) or saline, administered the night before ethanol gavage (2.5 g/kg). Blood samples were collected at 30, 90, 150, 270, and 330 min post-gavage. Sample sizes: *n* = 6 for ethanol (gavage) + saline group, *n* = 6 for ethanol (gavage) + GLP-1R agonist group. Statistical analysis by two-way ANOVA with Holm–Šídák’s test showed a significant *P* < 0.01 difference between the groups at times (30, 90, 150 min) with a mean difference of −29 (SE 7.8). This was performed on female mice. **G** Blood ethanol concentration over time following intraperitoneal (IP) ethanol injection (2.5 g/kg). Blood samples were collected at 30, 90, 150, 270, and 330 min post IP injection. Sample sizes: *n* = 6 for ethanol (IP) + saline group, *n* = 6 for ethanol (IP) + GLP-1R agonist group. Statistical analysis by two-way ANOVA with Holm–Šídák’s test showed a significant *P* < 0.001 difference between the groups at times (30, 90, and 150 min) with a mean difference of −70 (SE 13.4). This was performed on male mice.

It was expected, and we confirmed that with free access to ethanol, GLP-1R agonists would reduce ethanol intake, liver steatosis, inflammation and ROS. The reduction in ethanol intake provides a therapeutic rationale for the use of GLP-1R agonists in ALD. Since GLP-1R are absent, or are present only at very low levels on hepatocytes, it was also expected that in the absence of ethanol or nutrient regulation GLP-1R agonism would not affect ethanol-metabolizing enzymes. In fact, the opposite was discovered, and GLP-1R agonism was found to significantly reduce expression of hepatic *Cyp2e1* independently of nutrient and ethanol intake, with the predictable consequence of higher serum ethanol levels after a fixed ethanol dose. These findings have several real-world consequences. The hepatotoxicity of ethanol is primarily from metabolism to acetaldehyde, and by reducing ethanol metabolism from ethanol to acetaldehyde, GLP-1R agonism may be hepatoprotective, independent of a decrease in ethanol consumption^14^. This would broaden the rationale for the therapeutic use of GLP-1R agonism in ALD beyond individuals who have successfully reduced their ethanol intake. A second consequence of the use of GLP-1R agonists may be the higher-than-expected blood ethanol levels. The full implications for this have yet to be assessed. If the metabolism of ethanol in other organs is not reduced by GLP-1R agonists, then it is possible that the elevated blood levels caused by reduced hepatic metabolism could have detrimental effects on other organs, shifting ethanol metabolism and production of acetaldehyde from the liver to other organs. Finally, the mechanism by which central GLP-1R agonism can regulate hepatic gene transcription in the absence of GLP-1R on hepatocytes is of great interest. This may be due to GLP-1R-mediated upregulation of the sympathetic system, as has recently been demonstrated for central GLP-1R effects on innate immunity^15^.

## Methods (including statistics and reproducibility)

### Animals model

Animal studies were conducted according to guidelines set by the National Institutes of Health (NIH) and protocols approved by the Animal Care and Use Committee of Yale University. Mice were kept in individual ventilated cages.

First experiment: (Ethanol study), twenty-four male C57BL/6 N mice aged 12–14 weeks were purchased from NIAAA and were kept in an environment with controlled temperature (23˚C ± 1 °C) and a 12-h light/12-h dark cycle. They were given unrestricted access to both water and standard mouse chow after a pre-acclimation period of one week. Subsequently, the mice were acclimated to the Lieber-Decarli liquid control diet for five days, ad libitum. Half of the cohort were continued on the control diet while the other half were switched to an ethanol diet. Within both groups, mice were subdivided to receive subcutaneous semaglutide or saline for 15 days (Supplementary Fig. 1A). Thus, the groups were as follows: Group 1 (*n* = 4) - control liquid diet; Group 2 (*n* = 8) - ethanol diet; Group 3 (*n* = 8) - ethanol diet with subcutaneous semaglutide; and Group 4 (*n* = 4) - control diet with subcutaneous semaglutide.

Second experiment: (Fasting study), We obtained male and female C57BL/6 N mice aged 12–14 weeks, and they were given unrestricted access to both water and standard mouse chow after a pre-acclimatizing period of 1 week. The mice were separated into four groups. Group 1 (*n* = 4) – chow diet; Group 2 (*n* = 5) – chow diet with semaglutide; Group 3 (*n* = 8) 16-h fasting mice; and Group 4 (*n* = 7) 16-h fasting mice with semaglutide. The mice received a single dose of 30 nmol/kg of subcutaneous injection of semaglutide before fasting. After 16 h mice were euthanized, and liver tissues were collected.

A priori, it was established that any animal showing signs of illness or distress prior to the start of the experiment would be excluded from participation. Criteria for exclusion included weight loss greater than 10% of the group mean, reduced food or water intake, or lack of grooming behavior. No animals met these criteria, and thus none were excluded. The experimental procedures were performed by regulations adopted by the National Institute of Health and approved by the Institutional Animal Care and Use Committee, Yale University. Anesthesia was induced using isoflurane, and animals were sacrificed by cervical dislocation under isoflurane anesthesia.

### Diets

Chronic plus binge ethanol feeding was given as per the NIAAA protocol^16^. The Lieber-Decarli control liquid diet was prepared by combining 225 g of dry mix (Bio-Serv, Product F1259SP) with 860 ml of potable water to produce 1000 ml of control liquid diet. The ethanol liquid diet consisted of 133 g of dry mix (Bio-Serv, Product F1258SP), 20.3 g of maltose dextrin, 910 ml of potable water, and 52.6 ml of 95% ethanol, producing 1000 ml of ethanol liquid diet. For the maltose gavage, 9 g of maltose dextrin was dissolved in 20 ml of water. The ethanol gavage was prepared by mixing 6.6 ml of 95% ethanol with 13.4 ml of water to produce 20 ml of solution. Mice received gavage once on day 16. Body weight and food consumption were tracked daily. For the second experiment (Fasting study) mice on the chow arm were maintained on a standard chow diet, While the fasting arm mice have only access to the water for 16 h.

### GLP-1A administration

For the first experiment (ethanol study), mice in Groups 3 and 4 (semaglutide groups) received semaglutide (OZEMPIC; NDC Code: *0169-4130-01*) via subcutaneous injection every morning at 9:00 am for a period of 15 days post liquid diet acclimatization^12^. Semaglutide dose escalation was performed to a target dose of 30 nmol/kg as follows: 1 nmol/kg on days 1 and 2; 3 nmol/kg on days 3 and 4; 10 nmol/kg on days 5 and 6; and 30 nmol/kg on days 7 through 15. Semaglutide doses were based on published data^17^. The dose titration was performed to minimize GLP-1A-associated side effects. The other two groups were administered 0.9% sodium chloride injections to serve as a control for any potential stress due to the injection.

For the second experiment (Fasting study) a subgroup from the fasting arm and a subgroup from the chow arm received either single dose of semaglutide 30 nmol/kg or 0.9% sodium chloride injections to serve as control for stress due to injection.

### Sample collection

For the first experiment (ethanol study), on day 16, mice in Groups 2 and 3 (Lieber-Decarli ethanol diet groups) were gavaged with 5 g/kg of body weight ethanol, while mice in Groups 1 and 4 were gavaged with 9 g/kg of body weight maltose dextrin. At 9 h following gavage, mice were euthanized and blood samples and tissue samples were collected for histology, RNA and protein analyses. For histology, the left hepatic lobe was enclosed in a prelabelled histology cassette and fixed in 10% neutral buffered formalin for 24 h, followed by 70% ethanol for another 24 h before submission for tissue processing. Other portions of the liver were snap-frozen in liquid nitrogen until further processing.

For the second experiment (Fasting study): Mice were sacrificed after 16 h of fasting, and livers were collected and snap-frozen in liquid nitrogen until further processing.

### H&E and Immunofluorescent

Formalin-fixed liver tissues were processed for paraffin embedding and stained with hematoxylin and eosin. The slides were scanned using a Motic Easy Scan slide scanner at 40× objective setting and were assessed using the Motic DS assistant software. Paraffin sections were immunostained to detect PLIN2, 4HNE, and CYP2E1 expression. Briefly, the paraffin sections were cleared in xylene and hydrated in descending grades of ethanol. The slides were subjected to antigen retrieval via citrate buffer pH 6.5 (Vector Labs) using a heat retrieval protocol. The slides were then blocked with 2% goat serum in 1 × TBST for 1 h at room temperature, and incubated with anti-mouse PLIN2 antibody (Thermo Fisher Scientific Cat #15294-1AP), anti-mouse 4HNE antibody (Thermo Fisher Scientific Cat #MA5-27570) and anti-rabbit CYP2E1 antibody (Abcam Cat #ab28146). The recommended dilution was used overnight at 4 °C followed by staining with anti-rabbit/mouse antibody tagged with Alexa 555 with recommended dilution for 1 h at room temperature. The slides were washed and mounted with Prolong gold anti-fade mountant with DAPI (Thermo Fisher Scientific) and imaged using white light laser Leica SP8 confocal laser scanning microscope. The images were mostly acquired under a 40 × oil immersion objective with 1024 × 1024 pixel dimensions. Photomultiplier tube detectors were used for abundant proteins at visible range wavelengths. The images from immunostaining were exported as Leica image files and were processed using LASX software. The images were uniformly amended with minimal Gaussian blur to reduce graininess.

### Confocal microscopy

Fluorescence images of immunostained liver tissue sections were taken with a WLL (while light laser) Leica Stellaris DIVE confocal microscope. The images were mostly acquired under a 25× water immersion objective with 1024 × 1024 pixel dimensions. A highly sensitive HyD detector with gating was used for imaging. The images from immunostaining were exported as Leica image files (LIF) and were processed using IMARIS software version 9.8 (bitplane). The images were uniformly amended with minimal Gaussian blur to reduce graininess. The images of individual channels and/or overlay were saved as high-resolution TIF files. Further, the exported images were analyzed using the ImageJ software package (NIH) for further analysis.

### Quantitative real-time PCR

RNA was extracted from snap-frozen mouse liver samples. Approximately 10 mg of liver tissue was homogenized in Qiazol lysis buffer, and RNA was extracted using MiRNeasy Micro Kit (50) (QIAGEN Cat #74104). Following this, the RNA was transcribed into cDNA employing the oligo(dT) primer and the High-capacity cDNA Reverse Transcription Kit (Thermo Fisher Scientific Cat #4368814). Subsequently, quantitative RT-PCR was performed utilizing the Quant Studio 6 flex instrument (Thermo Fisher Scientific Cat #4485691). The expression of each gene of interest (Supplementary Table 1) was assessed using the Comparative CT method employing SYBR green master mix (Invitrogen Cat #4309155). The CT values of all the groups for expression of each gene of interest were normalized to their respective β-actin values and were presented as relative fold change of gene expression as compared to the control group.

### Western blot

The total protein extracts were prepared using RIPA buffer (50 mM Tris, 150 mM NaCl, 1% Triton X-100, 1% sodium deoxycholate, 0.1% sodium dodecyl sulfate (SDS), 1 mM phenylmethylsulfonyl fluoride (PMSF), 1 mM Na3VO4, 5 mM NaF, and 1% cocktail protein protease inhibitors (Sigma). Samples were processed on ice and then were centrifuged at 13,200×*g* for 10 min at 4 °C and 1X protease inhibitor in a microcentrifuge. The concentrations of protein extracts were determined by using the BCA Protein Assay Kit (Thermo Fisher Cat #23225), then protein samples were mixed with 3× loading buffer and loaded in equal amounts into the wells of 4–12% Bis-Tris protein gels (Bio-Rad) to transfer the proteins to PVDF membranes (Millipore).

The primary antibodies used for analysis included anti-CYP2E1 (1:1000; Abcam, Cat #ab28146), anti-β-Actin (1:1000; Cell Signaling, Cat # 4967), and anti-GAPDH (1:1000; Cell Signaling Technology, Cat #2118). Secondary horseradish peroxidase-conjugated antibodies (1:10,000; Cell Signaling Technology, Cat #7076 (mouse) and 7074 (rabbit) were used for analysis.

### Bulk RNA Seq

RNA quality was determined by RNA integrity number with Agilent Technologies 2100 Bioanalyzer (Cat #5067-1511), to confirm that all samples had an RNA integrity number exceeding 9 and were deemed suitable for further analysis. Total RNA (1 μg) was used for library synthesis using TruSeq V2 RNA-Seq kit. RNA sequencing library was prepared via the rRNA depletion method for the first experiment (ethanol study). Quality control analysis and quantification of the sequencing library were performed using Agilent Technologies 2100 Bioanalyzer High Sensitivity DNA Chip (Cat #5067-5592). Paired-end sequencing was performed on Illumina’s NovaSeq 6000 sequencing system. The library preparation and sequence analysis were performed by the Yale Center for Genome Analysis (YCGA), and our lab performed bioinformatics analysis.

### Pre-processing and differential gene expression analysis of bulk RNA-seq data

Quality check and alignment of the reads was done by Partek Flow; HISAT software was used to compare the reads against the mouse reference genome *Mus musculus* (mouse) – mm39; and mapped reads were obtained using HTSeq. The count matrix was loaded into RStudio (version 4.3.1), prefiltering of low reads (≤10 reads) was performed, and DESeq2 package version 1.42.0 was used to perform statistical analysis and normalize the DEGs between the groups, genes reaching statistical significance with adjusted *P* value <0.05 between the groups were examined. Over-representation analysis of the DEGs was done by using ShinyGo 0.80 to show the top 20 pathways, ranked based on fold enrichment, and false discovery rate (FDR ≤0.05).

### Blood ethanol concentration BAC experiment

We obtained male and female C57BL/6 N mice aged 12–14 weeks, and they were given unrestricted access to both water and standard mouse chow after a pre-acclimatizing period of 5 days. The mice were separated into two groups; one group was given a single subcutaneous injection of 30 nmol/kg b.wt. semaglutide or subcutaneous saline overnight. On the next day, mice were given a single dose of oral gavage of 2.5 g/kg b.wt. ethanol with a 20 G gavage needle (catalog no. AFN2038C) to both groups. Two additional groups were also injected intraperitoneal (IP) 2.5 g/kg b.wt. ethanol directly into the lower left area of the mouse abdomen with 25G needle and 1-ml syringe to eliminate the decreased gastric emptying effect of GLP-1RA. The experiments were conducted in three parts:

Experiment 1 (male mice): Ethanol (gavage) + saline group (*n* = 5) vs. ethanol(gavage) + GLP-1R agonist group (*n* = 4). One animal was excluded due to cessation of breath and death following alcohol gavage.

Experiment 2 (female mice): Ethanol (gavage) + saline group (*n* = 6) vs. ethanol(gavage) + GLP-1R agonist group (*n* = 6).

Experiment 3 (male mice, IP ethanol): Ethanol (IP) + saline group (*n* = 6) vs. ethanol (IP) + GLP-1R agonist group (*n* = 6) was done on male mice.

Whole blood samples were collected from the tail vein in 20-μl heparinized capillary tubes (Fisher Scientific company LLC #22-362566), anesthesia was induced using isoflurane, the blood samples were transferred quickly (10 s) to 2-ml gas chromatographic septum sealed vials (Sigma #29381-U) containing 200 μl of an internal standard solution consisting of 0.6 N perchloric acid and 4 mM n-propanol in double distilled water on ice. Vials were stored at 4 °C and analyzed the next day by headspace gas chromatography^18^.

Headspace analysis was performed by Yale Chemical and Biophysical Instrumentation Center (CBIC). The concentrations of EtOH samples were calculated by comparing the integrated areas of EtOH peaks (3 peaks) on the gas chromatograms with those of 1-propanol peaks (2 peaks), internal standards added in each sample and were normalized to the internal standard (1-propanol). A calibration curve was prepared with known EtOH concentrations and 1-propanol as the internal standard to calculate the absolute ethanol concentration in the blood samples.

### Statistical analysis

A randomization algorithm was used for randomly assigning each cage to the various experimental groups, while treatments were administered in a randomized order per cage. All cages were kept in the same row of each rack. To minimize bias, blinding was applied at various stages of the study. FZ was blinded during the allocation of the groups, conduct of the experiments, and outcome assessments, while AS, WM, and BB were not. All investigators were involved in the data analysis stage were not blinded during this phase. The sample size for the experiments was based on previous experience in the lab with a similar model.

All statistical analyses were performed using Prism 10.0.2 by GraphPad Software, Inc. (CA, USA). Data on ethanol consumption and body weight were analyzed using one-way ANOVA, followed by Šídák’s multiple comparisons. qPCR results from the first experiment (ethanol study) were analyzed using one-way ANOVA, followed by Dunnett’s test. Immunofluorescent quantifications were analyzed by Two-way ANOVA, followed by post-hoc test (Holm–Šídák’s). qPCR and western blot results from the Second experiment (Fasting study) were analyzed using the Mann–Whitney *U*-test. Western blot results from the first experiment (ethanol study) were analyzed by Brown–Forsythe and Welch’s ANOVA tests.

Two-way repeated measures ANOVA was used to compare blood ethanol concentration between the two groups over the five-time points, considering group and time as factors. Follow up with post-hoc (Holm–Šídák’s). Analyses were done separately for each experiment based on sex and route of drug administration.

## Supplementary information

Supplementary information

## Data Availability

Bulk RNA sequencing data from the first experiment (Alcohol study) has been deposited in the Gene Expression Omnibus (GEO) under accession number GSE291407. All relevant raw and processed data are included and will be publicly available upon publication.
