# Supplementary material for: GLP-1 receptor agonism results in reduction in hepatic ethanol metabolism
Source: NPJ Metab Health Dis. 2025 Sep 18;3:36. doi: 10.1038/s44324-025-00077-y (PMC12446429; doi:10.1038/s44324-025-00077-y)
Supplement: Supplementary file 1 — Supplementary information [file 44324_2025_77_MOESM1_ESM.pdf]

Supplemental Table 1.

Gene Name	Primer
**Nox2**	(TGGCGATCTCAGCAAAAGGTGG, GTACTGTCCCACCTCCATCTTG)
**Fasn**	(CACAGTGCTCAAAGGACATGCC, CACCAGGTGTAGTGCCTTCCTC)
**Il6**	(TACCACTTCACAAGTCGGAGGC, CTGCAAGTGCATCATCGTTGTTC)
**Nrf2**	(CAGCATAGAGCAGGACATGGAG, GAACAGCGGTAGTATCAGCCAG)
**Mpo**	(CGTGTCAAGTGGCTGTGCCTAT, AACCAGCGTACAAAGGCACGGT)
**Sreb1f**	(CGACTACATCCGCTTCTTGCAG, CCTCCATAGACACATCTGTGCC)
**Il12**	(ACGAGAGTTGCCTGGCTACTAG, CCTCATAGATGCTACCAAGGCAC)
**Acc1**	(GTTCTGTTGGACAACGCCTTCAC, GGAGTCACAGAAGCAGCCCATT)
**Cyp2e1**	(AGGCTGTCAAGGAGGTGCTACT, AAAACCTCCGCACGTCCTTCCA)

Supplemental Table 1: Primer sequence of the genes of interest.

Supplemental Table 1.

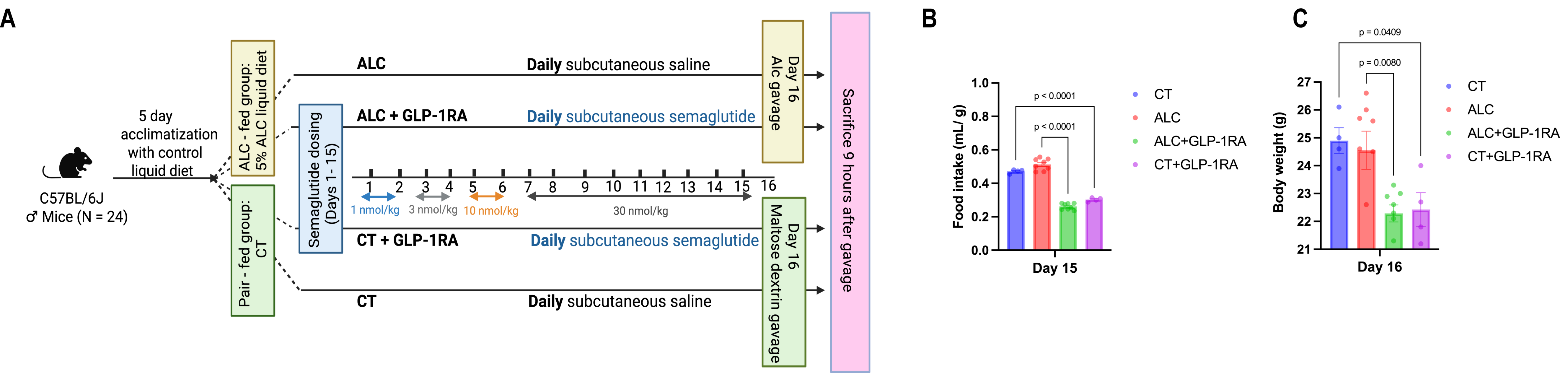

Figure Supplementary 1: GLP-1R agonism resulted in reduced food intake and ethanol consumption. **(A)** Experimental setup: following a 5-day acclimatization, 24 mice were divided into four groups—1) Control (CT), 2) Ethanol-fed (ALC), 3) Ethanol + GLP-1R agonist (ALC + GLP-1R), 4) Control + GLP-1R agonist (CT + GLP-1R). The semaglutide dosage regimen was escalated from 1 to 30 nmol/kg over 16 days. On day 16, mice in the ethanol groups received ethanol gavage with 5g/kg of body weight, and control groups received dextrin 9 h following gavage, mice were euthanized .

(B) Comparison of food intake between groups on day 15. Data are shown as mean ± SD, with statistical analysis by one-way ANOVA with Holm-Šídák's test.

(C) Comparison of body weight between groups on day 16. Data are shown as mean ± SD, with statistical analysis by one-way ANOVA with Holm-Šídák's test.

Figure Supplementary 2.

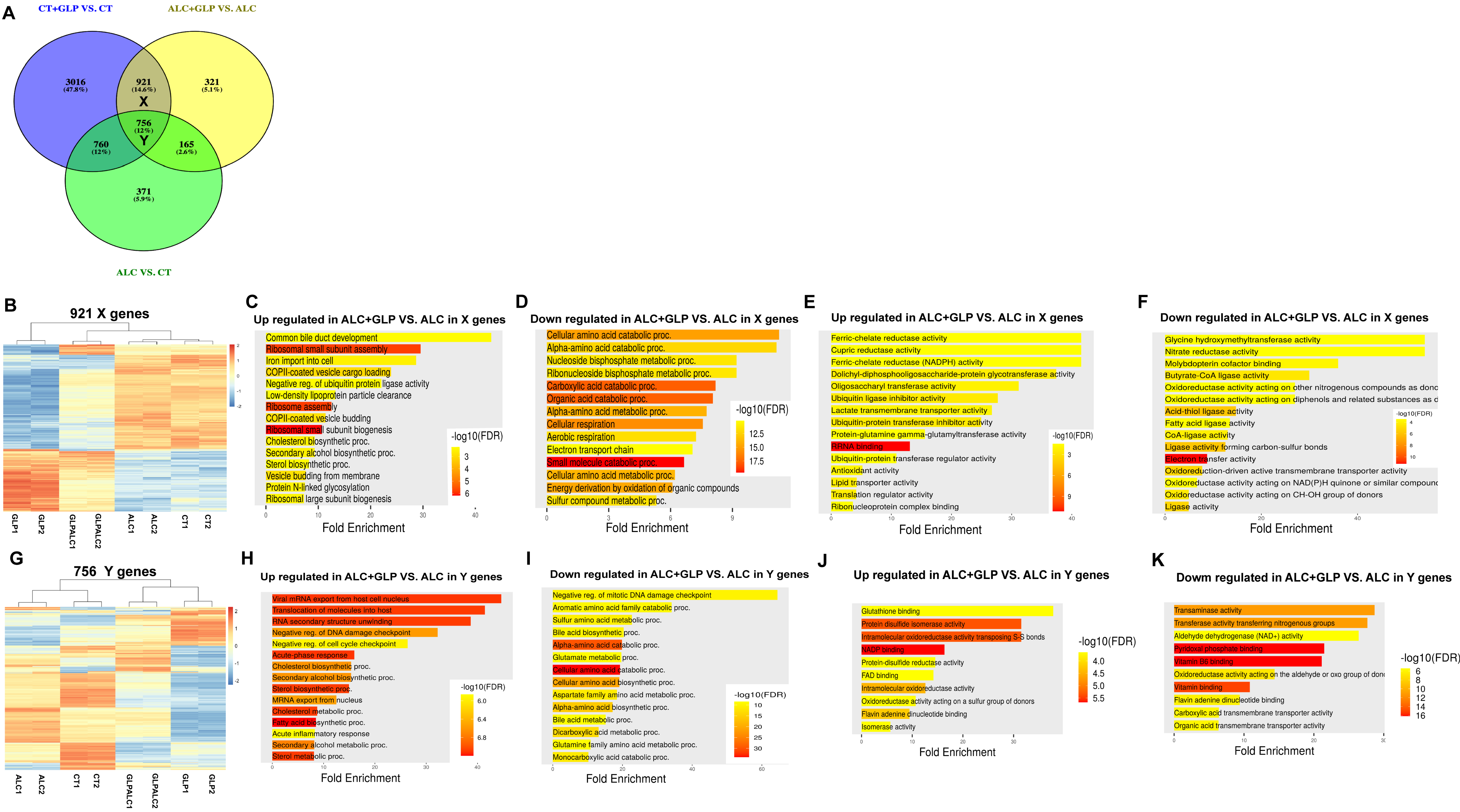

Figure Supplementary 2: GLP-1R Agonism Results in a Reduction in Many Genes Involved in Ethanol Metabolism.
(A) Venn diagram showing significant DEGs (p-adjusted < 0.05) across group comparisons: control + GLP-1R agonist vs. control alone (blue), ethanol + GLP-1R agonist vs. ethanol alone (yellow), and ethanol vs. control alone (green).
(B) Heatmap of the 921 significant DEGs (p-adjusted < 0.05) DEGs identified in the crossover region X of the Venn diagram.
(C-F) Enrichment analysis for molecular functions (MF) associated with DEGs in the ethanol + GLP-1R agonist vs. ethanol alone comparison in genes (X).

(G) Heatmap of the 756 significant DEGs (p-adjusted < 0.05) DEGs identified in the Venn diagram.
(H-I) enrichment analysis for molecular functions (MF) associated with DEGs in the ethanol + GLP-1R agonist vs. ethanol alone comparison in genes (Y).

(J-K) enrichment analysis for biological process (BP) associated with DEGs in the ethanol + GLP-1R agonist vs. ethanol alone comparison in genes (Y).

Figure Supplementary 3:

A B C

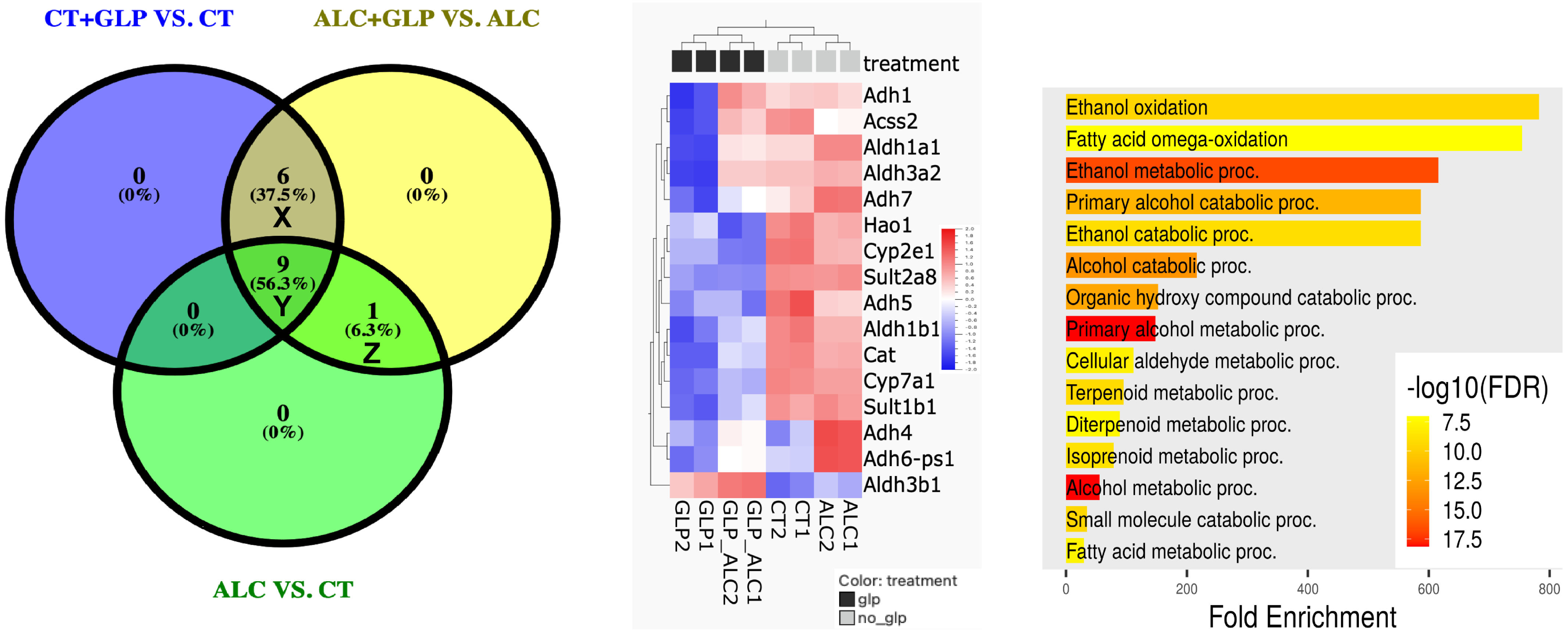

D

X Genes	Y Genes	Z Genes
Ald3b1	*Aldh3a2*	*Adh4*
Hao1	*Cyp2e1*	
Sult1b1	*Cat*	
Sult2a8	*Acss2*	
Aldh1b1	*Adh5*	
Adh1	*Cyp7a1*	
	Aldh1a1	
	Adh7	
	Adh6-ps1	

Figure Supplementary 3: Ethanol Oxidation and Catabolism Related Genes are Regulated by Ethanol and by GLP-1R Agonism.

(A) Venn diagram of the significant (p-adjusted <0.05) DEGs involved in ethanol oxidation and catabolic processes, with comparisons as follows: control + GLP-1R agonist vs. control alone (blue), ethanol + GLP-1R agonist vs. ethanol alone (yellow), and ethanol vs. control alone (green).
(B-C) Heatmap of the significant (p-adjusted <0.05) genes associated with ethanol oxidation and catabolic processes, enriched for pathways based on the Kyoto Encyclopedia of Genes and Genomes (KEGG), with p-adjusted < 0.05.

(D) Distribution and the list of the significant (p-adjusted <0.05) DEGs (X, Y, Z) of ethanol oxidation and catabolic processes in the three comparisons.
